# Novel water-based automated endoscope cleaning process vs conventional manual cleaning for reducing duodenoscope contamination

**DOI:** 10.1055/a-2536-8061

**Published:** 2025-03-14

**Authors:** Koen van der Ploeg, Juliëtte A. Severin, Margreet C. Vos, Nicole S Erler, Adriana J.C. Bulkmans, Marco Bruno, Bibi C.G.C. Mason-Slingerland

**Affiliations:** 1 6993 Department of Gastroenterology and Hepatology, Erasmus MC, Rotterdam, Netherlands; 2 6993 Department of Medical Microbiology and Infectious Diseases, Erasmus MC, Rotterdam, Netherlands; 3 6993 Department of Biostatistics, Erasmus MC, Rotterdam, Netherlands; 4 6993 Quality Assurance and Regulatory Affairs office Medical Technology, Erasmus MC, Rotterdam, Netherlands

**Keywords:** Pancreatobiliary (ERCP/PTCD), ERC topics, Quality and logistical aspects, Quality management, Hygiene

## Abstract

**Background and study aims:**

Duodenoscope contamination remains a persistent problem, exposing patients to infection risks. Automation in reprocessing may limit human error, reduce workload, and increase uniformity and traceability. However, its effectiveness should be evaluated before implementation. This study assessed the impact of implementing a novel water-based automated endoscope cleaning process on duodenoscope contamination.

**Methods:**

This before-and-after intervention study compared duodenoscope cleaning methods. From January 2022 to December 2023, conventional manual cleaning was used. From January 2024 to June 2024, the AquaTYPHOON system (AT) replaced manual cleaning. Cultures from Pentax ED34-i10T2 patient-ready duodenoscopes were collected. The main outcome was the contamination rate with microorganisms of gut or oral origin (MGO). Secondary outcomes included contamination with solely gut bacteria. Non-inferiority of the AT was tested using a generalized estimating equation with a non-inferiority margin of 5%.

**Results:**

During the manual cleaning period, 333 duodenoscope cultures of eight duodenoscopes were collected; during the AT period, 100 cultures were collected. Pre-introduction of the AT, the contamination rate with MGO was 21.6%, which fell to 16% post-introduction (risk difference: –5.6%, upper bound 90% confidence interval [CI] 6.8%). For gut bacteria, the contamination rate decreased from 14.4% to 9% (risk difference: –5.4%, upper bound 90% CI 3.9%), indicating non-inferiority.

**Conclusions:**

AT reduced the contamination rate with MGO, but non-inferiority was not demonstrated. For gut bacteria, AT was non-inferior to manual cleaning. These results are promising. However, future studies should confirm these findings in larger samples and explore other advantages of using the AT in duodenoscope cleaning.

## Introduction

During endoscopic retrograde cholangiopancreatography (ERCP) procedures, duodenoscopes are heavily exposed to gastrointestinal bacteria. Consequently, these devices must undergo comprehensive reprocessing to prevent patient-to-patient pathogen transmission 1 2 . However, reprocessing may fall short, with microorganisms persisting in up to 15% of cases 3 . Manual cleaning, crucial for removing organic debris before automated cleaning and disinfection, involves brushing and flushing duodenoscope channels with water and a detergent solution 4 5 . However, healthcare workers often face time pressure, complex protocols, and physical discomfort during manual cleaning 6 7 . Surveillance studies by the U.S. Food and Drug Administration showed that more than 60% of manual cleaning tasks are improperly executed 8 .

Automated reprocessing could improve endoscope cleaning pre-disinfection by standardizing practices, alleviating staff workload, and minimizing human errors 9 . The recently introduced AquaTYPHOON system (AT, Plasmabiotics; Pentax Medical), which employs pulsating high-velocity water and compressed air instead of brushes and detergent, shows promising preliminary validation results in clinical settings 10 . Gastrointestinal endoscopes of all types were cleaned with the AT and validated by demonstrating compliance with the defined target level of a residual bacterial load of ≤ 6 colony-forming units (CFU) after reprocessing, as well as protein removal in accordance with ISO standards 15883–4 and 15883–5 10 . However, guidelines recommend that any CFUs of gastrointestinal microorganisms warrant endoscope quarantine 11 12 . Because the types of microorganisms detected were not mentioned, true validation of the AT has not been demonstrated. Moreover, the studies report that the AT has been validated across multiple endoscope types from major endoscope manufacturers. However, the number of duodenoscopes included in the validation studies is unclear. Because most reported endoscope-associated outbreaks originate from contaminated duodenoscopes, this must be confirmed prior to implementation 10 13 . This study aimed to show non-inferiority of the AT in patient-ready duodenoscopes compared with conventional manual cleaning methods.

## Methods

### Setting

This retrospective-prospective before-and-after single-center intervention study was conducted between January 2022 and June 2024 at the Erasmus University Medical Center (Erasmus MC), a large Dutch tertiary care center that performs approximately 750 ERCP procedures annually.

### Data collection

Between January 2022 and December 2023, duodenoscope cultures from Pentax ED34-i10T2 duodenoscopes with disposable endcaps were collected as part of the PREVENT study (unpublished). During this period, reprocessing was performed according to manufacturer instructions, and the protocols have been described previously 14 15 . The cultures collected during this period were stored in a database. For the purpose of this study, we excluded cultures from loaner duodenoscopes that were no longer in active use. The remaining cultures from this database were used to represent the first study period, during which conventional manual cleaning methods, including flushing and brushing, were employed. These data were compared with cultures collected in the subsequent prospective period, during which the AT was implemented, as described below.

### Intervention

**Fig. 1:**
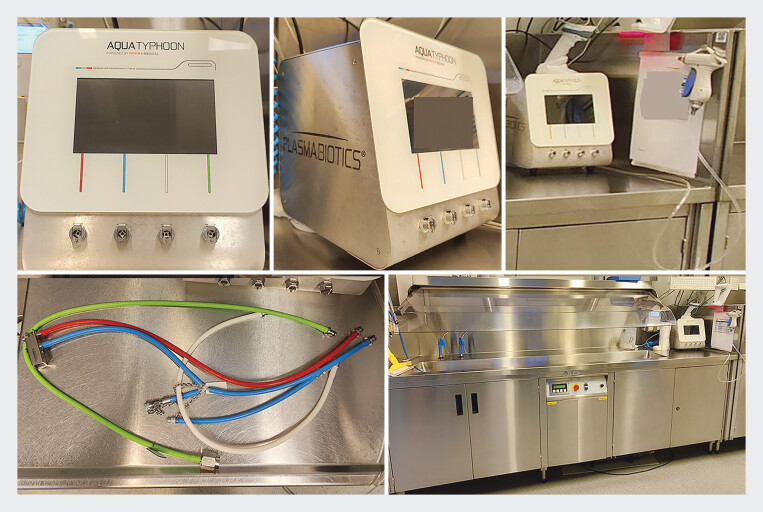
Illustrations of the AquaTYPHOON system, showing the device's front and side views (top left and center), the barcode scanner and AquaJET (top right), color-coded endoscope connection tubes (bottom left), and the cleaning sink (bottom right).

The AT includes the AquaTYPHOON device, a barcode scanner, a water pistol (AquaJET), and a label printer ( Fig. 1 ). The automated process includes a leak test and uses high-velocity air and water to create a turbulent flow in the endoscope channels to remove organic material and debris. During the cleaning process, the duodenoscope is not submerged in water and no detergents are used. The AquaJET is used by reprocessing personnel to clean the endoscope externally. The AquaTYPHOON device displays the current phase of the process and its percentage of completion. The time needed for the AT to complete automated cleaning is 5 minutes. If an error occurs, such as low air pressure, the cycle is interrupted and the error is displayed. Data records are printed and stored on the device. In this study, the printer was not used.

After the AT received its CE mark in March 2023, we developed the protocol for this study and the non-inferiority margin, endpoints, and analysis plan were specified (detailed below). In January 2024, all duodenoscopes were inspected using a borescope and those requiring maintenance were sent to the manufacturer for repairs. When the duodenoscopes returned from the manufacturer, they were included in the study. Before transitioning to the AT period, reprocessing personnel received training from the manufacturer on use and maintenance of the system. In addition, all duodenoscopes underwent four consecutive reprocessing cycles using the AT, followed by automated cleaning and disinfection as a wash-out measure. These four cycles were performed sequentially, without any intermittent use in a patient, and the duodenoscopes were confirmed to be culture-negative before the start of the AT period. Furthermore, all duodenoscopes that returned from the manufacturer were cultured prior to reintroduction for clinical use. The process of bedside precleaning, automated cleaning, high-level disinfection (HLD), and drying remained unchanged 15 . Duodenoscope cultures during the AT period were collected between January 2024 and June 2024.

### Sampling

Sampling of the duodenoscope was performed shortly before its use for an ERCP procedure. Initially, the distal tip of the duodenoscope was swabbed using a dry Copan Liquid Amies Elution Swab (eSwab Copan). To neutralize any residual disinfectants, 1 mL of a neutralizer (Dey-Engley broth) was introduced into the container. Subsequently, the suction and biopsy channels were subjected to a combined flush-brush-flush procedure that encompassed the entire length of the duodenoscope, from the umbilical connector (processor end) to the distal tip. This involved flushing each channel with 20 mL of sterile water, which was collected in a sterile container containing 40 mL of neutralizer. A single-use endoscope cleaning brush (CS5522A, Pentax) was then passed through the entire length of the channels and the distal tip of the brush was severed using sterile scissors and added to the container. Flushing of both channels was then repeated to complete the sampling process.

### Microbiological protocols and interpretation

Microbiological methods used were as previously reported 4 . Contamination was defined as: 1) ≥ 1 CFU of microorganisms of gut or oral origin (MGO), including *Pseudomonas aeruginosa* , *Staphylococcus aureus* , and yeasts; or 2) ≥ 20 CFU/20 mL of any microorganism of other origin (AM20) 16 . Culture results used in this study were collected separately from routine microbiological surveillance conducted monthly at Erasmus MC. Consequently, duodenoscopes contaminated with MGO were not quarantined. However, if cultures from routine surveillance tested positive for MGO, the duodenoscopes were quarantined according to Dutch guidelines 12 . These duodenoscopes were cleared for clinical use only after subsequent negative culture results.

### Outcomes

The primary endpoint was the proportion of duodenoscope cultures positive for MGO. Secondary endpoints included the proportion of duodenoscope cultures positive for gut, oral, and AM20 microorganisms.

### Sample size determination

Sample size calculation was based on the database of duodenoscope cultures from the PREVENT study, which at that time contained 270 culture results from the period using conventional cleaning. The contamination rate for conventional cleaning was approximately 20%. Given implementation of the AT and considering outcomes from validation studies, we anticipated a contamination rate of 10%, making a 10% reduction in contamination a realistic expectation. We conducted a power analysis using simulation, with data simulated through a random intercept logistic mixed model. This model was based on assumptions about contamination rates from logistic mixed models fitted to the available duodenoscope culture results from conventional cleaning. We assumed that the random intercept standard deviation would remain consistent across different phases of the study.

The risk-difference of contamination with MGO between conventional cleaning and AT cleaning was assessed using a generalized estimating equation (GEE) to consider clustering due to repeated measurements of the same duodenoscopes. MGO-positivity served as the response variable, the study phase was the sole covariate, and the model used a Gaussian distribution with identity link. Our simulations suggested that with an expected contamination rate for the AT period of 10%, a sample of 100 duodenoscope cultures in the AT period would achieve approximately 80% statistical power.

Because there was a possibility that the contamination rate might increase after implementation of the AT, an interim analysis was performed after 50 duodenoscope cultures were collected during the AT period. To ensure patient safety, if use of the AT had resulted in significantly higher contamination rates compared with the conventional cleaning method, the study would have been terminated prematurely. Specifics regarding the analysis and threshold for early termination are described below. Because the interim analysis was conducted solely for futility monitoring without formal hypothesis testing for non-inferiority, no adjustments for multiplicity were deemed necessary.

### Statistical analyses

Statistical analyses were performed in R version 4.1.3 17 . Categorical variables are presented as counts or proportions (%), whereas continuous variables are described using the median with the first and third quartiles (Q1, Q3) or the mean and standard deviation (SD). The analysis was conducted on an intention-to-treat basis, including all duodenoscope cultures collected during the study periods, except those from loaner duodenoscopes no longer in active use. The primary analysis used a GEE model to assess differences in contamination rates between study phases. This model was the same as that used for sample size calculation. To determine non-inferiority of the AT, the upper limit of the two-sided 90% confidence interval (CI) for the parameter estimate of the study phase was compared with a margin of 5%. We chose a 90% CI, so that, due to its one-sided use in the non-inferiority test, the corresponding significance level is 0.05.

A futility interim analysis was conducted after collecting 50 duodenoscope cultures during the AT period. The same GEE model described above was utilized, except for a one-sided inferiority test that compared the lower bound of the 90% CI for the risk difference with a specified threshold for early termination of 5% in case the AT performed considerably worse than conventional manual cleaning.

## Results

### Culture characteristics

A total of 433 cultures were collected from eight Pentax ED34-i10T2 duodenoscopes. During the period when conventional manual cleaning was employed (January 2022 to December 2023), 333 duodenoscope cultures (76.9%) were collected, and 100 cultures (23.1%) were collected during the AT period (January 2024 to May 2024). Table 1 presents an overview of culture characteristics. During the period employing conventional cleaning methods, 21.6% of cultures (72/333) were contaminated with MGO, of which 48 (14.4%) contained gut bacteria and 26 (7.8%) contained oral bacteria. Of 100 cultures collected during the AT period, 16% (16/100) were contaminated with MGO, of which nine (9%) contained gut flora and eight (8%) contained oral flora. **Supplementary Table 1** in the supplementary appendix presents contamination rates of individual duodenoscopes according to each contamination definition.

**Table:** **Table 1** Culture characteristics of periods employing conventional cleaning methods compared with the AquaTYPHOON system.

	Conventional cleaning n = 333 (100%)	AquaTYPHOON system n = 100 (100%)
Microorganisms of gut or oral origin, n (%)	72 (21.6%)	16 (16.0%)
Gut, n (%)	48 (14.4%)	9 (9.0%)
Oral, n (%)	26 (7.8%)	8 (8.0%)
AM20, n (%)	303 (91.0%)	94 (94.0%)
Sampled Pentax ED34-i10T2 duodenoscopes, n (%)		
A110077	35 (10.5%)	16 (16.0%)
A110095	45 (13.5%)	10 (10.0%)
A110096	54 (16.2%)	6 (6.0%)
A110098	33 (9.9%)	21 (21.0%)
A110100	48 (14.4%)	22 (22.0%)
A110280	36 (10.8%)	8 (8.0%)
A110377	39 (11.7%)	17 (17.0%)
A110409	43 (12.9%)	0 (0.0%)
AM20, 20 CFU/mL of any other microorganism; SD, standard deviation.		

### Interim analysis

After 50 duodenoscope cultures were collected during the AT period, an interim analysis was performed. Six of 50 duodenoscope cultures (12%) were positive for MGO. The risk difference between the two periods was -9.3% (lower bound 90% CI -24.3%) in favor of the AT. Because the lower bound of the 90% CI was -24.3%, and thus less than the futility margin of 5%, the study was continued.

### Cultured microorganisms

**Fig. 2:**
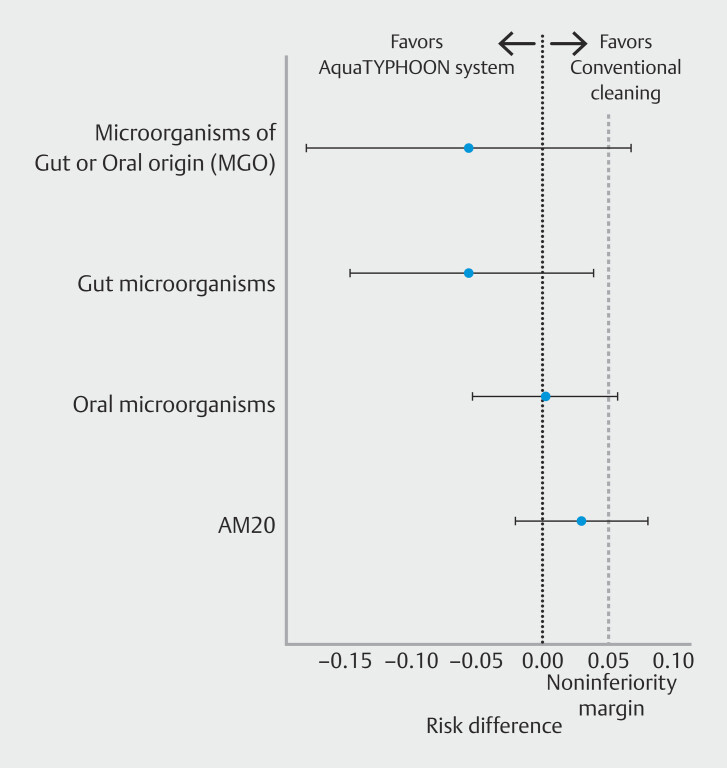
Forest plot with risk differences in contamination between the AquaTYPHOON system and conventional manual cleaning methods according to the different definitions. AM20, microbial growth with ≥ 20 colony-forming units/20 mL of water and/or skin type microorganism; MGO, presence of microorganisms of gut or oral origin.

Table 2 shows all cultured MGOs and their frequencies and all other cultured microorganisms are shown in the supplementary appendix ( **Supplementary Table 2** and **Supplementary Table 3** ). No contamination with *P. aeruginosa* , *Klebsiella pneumoniae* , or *Enterobacter cloacae* complex occurred during the AT period. However, contamination with *Stenotrophomonas maltophilia* occurred more frequently, seven times versus once in the conventional manual cleaning group. In the primary analysis, the risk difference in MGO-positivity between the AT and conventional manual cleaning methods was -5.6% (upper bound 90% CI 6.8%) ( Fig. 2 ). For contamination with gut bacteria, the risk difference was -5.4% (upper bound 90% CI 3.9%), and for oral flora, it was 0.2% (upper bound 90% CI 5.7%). For contamination with AM20, the risk difference was 3% (upper bound 90% CI 8.1%) in favor of conventional manual cleaning. Non-inferiority could not be demonstrated for contamination with MGO, oral flora, or AM20 because the upper bound of the 90% CI exceeded the 5% non-inferiority margin. However, non-inferiority could be demonstrated for contamination with gut bacteria.

**Table:** **Table 2** Cultured microorganisms of gut or oral origin.

	Conventional cleaning n = 333 (100%)	AquaTYPHOON system n = 100 (100%)
Gut bacteria, n (%)		
*Pseudomonas aeruginosa*	23 (6.9%)	0 (0.0%)
*Klebsiella pneumoniae*	16 (4.8%)	0 (0.0%)
*Stenotrophomonas maltophilia*	1 (0.3%)	7 (7.0%)
*Enterobacter cloacae* complex	5 (1.5%)	0 (0.0%)
*Escherichia coli*	2 (0.6%)	1 (1.0%)
*Klebsiella oxytoca*	2 (0.6%)	0 (0.0%)
*Hafnia alvei*	2 (0.6%)	0 (0.0%)
*Enterococcus faecium*	1 (0.3%)	0 (0.0%)
*Citrobacter koseri*	1 (0.3%)	0 (0.0%)
*Enterococcus faecalis*	1 (0.3%)	0 (0.0%)
*Acinetobacter iwoffi*	0 (0.0%)	1 (1.0%)
*Candida* species	1 (0.3%)	0 (0.0%)
*Paeniclostridium* species	1 (0.3%)	0 (0.0%)
*Staphylococcus aureus*	1 (0.3%)	0 (0.0%)
Oral bacteria, n (%)		
*Moraxella osloensis*	16 (4.8%)	4 (4.0%)
*Moraxella* species	6 (1.8%)	2 (2.0%)
*Actinomyces oris*	2 (0.6%)	0 (0.0%)
*Neisseria* species	1 (0.3%)	0 (0.0%)
*Neisseria subflava*	1 (0.3%)	0 (0.0%)
*Rothia mucilaginosa*	1 (0.3%)	0 (0.0%)
*Rothia* species	1 (0.3%)	0 (0.0%)
*Streptococcus mitis*	0 (0.0%)	1 (1.0%)
*Streptococcus sanguinis*	1 (0.3%)	0 (0.0%)
*Rothia dentocariosa*	0 (0.0%)	1 (1.0%)
*Streptococcus cristatus*	0 (0.0%)	1 (1.0%)

### Environmental sampling

Because of the unexpected increase in contamination with *S. maltophilia* during the AT period, 13 environmental samples from the endoscope reprocessing department were collected to check for a possible common source of contamination. The AquaTYPHOON device connectors, touchscreen, and water pistol were swabbed, as well as the automated endoscope reprocessors (AERs) and AER connectors, drying cabinet connectors, and a hand alcohol dispenser. All samples were negative for *S. maltophilia* . The drain of the sink where the endoscopes were cleaned was positive for *S. maltophilia* . However, this was not considered a likely source of the contamination.

## Discussion

After introduction of the AT to replace conventional manual cleaning, the MGO contamination rate of patient-ready duodenoscopes declined by 5.6% (from 21.6% to 16%). This finding illustrates that automated cleaning methods can improve the outcome of duodenoscope reprocessing in a real-world clinical setting. This effect was predominantly caused by a reduction in gut bacteria isolated from duodenoscope samples, which are known to be notorious for causing duodenoscope-associated infections. Total absence of *P. aeruginosa* and *K. pneumoniae* during the AT period is particularly promising because these bacteria are frequently involved in outbreaks caused by contaminated duodenoscopes 13 .

Enhancing automation in endoscope reprocessing offers numerous benefits, including improved efficiency, prevention of human error, standardization of procedures, and full traceability. Introduction of AERs in cleaning and HLD has been shown to improve guideline adherence, reduce physical discomfort associated with reprocessing, and decrease need for manual labor 9 . The same benefits may apply to automating manual cleaning. In addition, automated cleaning could significantly reduce the cognitive load imposed by manual reprocessing protocols 6 .

Manual cleaning and the AT system differ notably in their cleaning methodologies. Unlike manual cleaning, the AT system relies solely on water, without use of detergents or single-use brushes. In addition, the duodenoscope is not submerged during cleaning. These differences may reduce the environmental impact of reprocessing by eliminating detergent use and single-use components while potentially simplifying the cleaning process. However, incorporating submersion in water with detergents could further enhance AT effectiveness by breaking down organic residues on the outside of the endoscope. Future research should explore this as a potential modification of the cleaning process. Moreover, a comprehensive life cycle analysis and environmental impact assessment are necessary to determine the duration of AT use required to achieve a meaningful environmental benefit.

During the AT period, incidence of cultures contaminated with *S. maltophilia* increased, making it the most prevalent MGO contaminant. *S. maltophilia* is an opportunistic pathogen present in the gut, but also widely distributed in various environments, including water and soil. Gut colonization with *S. maltophilia* can particularly occur after patients have used broad-spectrum antibiotics. Given its resistance profile and ability to cause severe infections in vulnerable populations, *S. maltophilia* is considered an important nosocomial pathogen and it has been involved in outbreaks linked to contaminated bronchoscopes 18 19 20 . Bacteria that thrive in moist environments and readily form biofilms are prone to contaminating the AT. Therefore, the AT should be regularly monitored for contamination. Although the sudden increase in contamination prevalence with *S. maltophilia* suggested a common source of contamination other than a patient, such as contaminated AquaTYPHOON device connectors or AER, the environmental cultures did not show an external contamination source. However, *S. maltophilia* was cultured from the sink drain at the location where the AT was use. Because the duodenoscope was not submerged in water during cleaning with the AquaJET, we cannot completely exclude the possibility that splashes from the sink drain contaminated the duodenoscopes during cleaning.

Implementation of the AT led to an increase in contamination with AM20 by 3% (upper bound 90% CI 8.1%). In our previous research, we hypothesized that the high contamination rate with AM20 might be partly explained by the design of the single-use brush with a distal sweeper used in our center during manual cleaning 4 . However, even absent any materials introduced into the duodenoscope channels during precleaning, the contamination rate with AM20 did not decrease. The biomatrix of non-MGO bacteria might protect MGOs during HLD, preventing proper disinfection 21 . Thus, the source of such persistent contamination by AM20 remains unknown and requires further investigation.

This study is subject to certain limitations that could have impacted the results. This was a single-center study employing a single type of duodenoscope, which limits generalizability of our findings. In addition, due to our limited sample size, the CI of the main outcome was quite large, preventing us from showing non-inferiority despite a clear difference in the contamination rate with MGO. Furthermore, the order of the periods (conventional cleaning versus AT) was not randomized and no control group was available; therefore, we cannot claim causality between introduction of the AT and reduction in contamination with MGO. Finally, sampling and culturing of the air/water channel was not performed, leaving the effectiveness of the AT in cleaning this channel unestablished.

## Conclusions

In conclusion, this study found that implementation of the AT led to a reduction in contamination with MGO in Pentax ED34-i10T2 duodenoscopes, although non-inferiority was not demonstrated. However, the AT was non-inferior to conventional manual cleaning in reducing contamination with gut microorganisms. Therefore, the AT could offer an alternative to conventional manual cleaning methods. Future larger studies are necessary to confirm our findings, demonstrate generalizability, and investigate other potential benefits regarding reprocessing efficiency, labor intensity, and environmental impact.

## Supplementary Material

Supplementary Material Zusatzmaterial
