# Supplementary material for: Novel water-based automated endoscope cleaning process vs conventional manual cleaning for reducing duodenoscope contamination
Source: Endosc Int Open. 2025 Mar 14;13:a25368061. doi: 10.1055/a-2536-8061 (PMC11922170; doi:10.1055/a-2536-8061)
Supplement: Supplementary file 1 — Supplementary Material [file 10-1055-a-2536-8061_25382206.pdf]

Supplementary Table 1 Duodenoscope culture results per individual duodenoscope.

Sampled Pentax ED34-i10T2 duodenoscopes	MGO	Gut	Oral	AM20
**Conventional cleaning, N = 333 (100%)**				
A110077	3 (0.9%)	1 (0.3%)	2 (0.6%)	34 (10.2%)
A110095	8 (2.4%)	4 (1.2%)	4 (1.2%)	41 (12.3%)
A110096	12 (3.6%)	10 (3.0%)	2 (0.6%)	51 (15.3%)
A110098	2 (0.6%)	2 (0.6%)	0 (0.0%)	31 (9.3%)
A110100	11 (3.3%)	8 (2.4%)	4 (1.2%)	42 (12.6%)
A110280	1 (0.3%)	0 (0.0%)	1 (0.3%)	35 (10.5%)
A110377	10 (3.0%)	3 (0.9%)	7 (2.1%)	35 (10.5%)
A110409	25 (7.5%)	20 (6.0%)	6 (1.8%)	34 (10.2%)
**AquaTYPHOON system, N = 100 (100%)**				
A110077	3 (3.0%)	1 (1.0%)	2 (2.0%)	16 (16.0%)
A110095	0 (0.0%)	0 (0.0%)	0 (0.0%)	10 (10.0%)
A110096	4 (4.0%)	4 (4.0%)	1 (1.0%)	4 (4.0%)
A110098	5 (5.0%)	2 (2.0%)	3 (3.0%)	19 (19.0%)
A110100	2 (2.0%)	1 (1.0%)	1 (1.0%)	21 (21.0%)
A110280	1 (1.0%)	1 (1.0%)	0 (0.0%)	8 (8.0%)
A110377	1 (1.0%)	0 (0.0%)	1 (1.0%)	16 (16.0%)

AM20, 20 CFU/mL of any other microorganism; MGO, microorganisms of gut or oral origin.

Supplementary Table 2 Water type microorganisms cultured from duodenoscopes in the period employing conventional cleaning compared with the period employing the AquaTYPHOON system.

Water type microorganisms	Conventional cleaning methods (N = 333)	AquaTYPHOON system (N = 100)
Microbacterium species	104 (31.2%)	45 (45.0%)
Chryseobacterium species	93 (27.9%)	10 (10.0%)
Ochrobactrum intermedium	35 (10.5%)	21 (21.0%)
Gram-negative rods	34 (10.2%)	1 (1.0%)
Paracoccus yeei	22 (6.6%)	1 (1.0%)
Methylobacterium species	8 (2.4%)	9 (9.0%)
Microbacterium oxydans	2 (0.6%)	11 (11.0%)
Sphingobacterium spiritivorum	9 (2.7%)	2 (2.0%)
Pseudoxanthomonas mexicana	8 (2.4%)	0 (0.0%)
Rhizobium radiobacter	5 (1.5%)	2 (2.0%)
Microbacterium paraoxydans	1 (0.3%)	3 (3.0%)
Ochrobactrum species	3 (0.9%)	1 (1.0%)
Sphingomonas paucimobilis	0 (0.0%)	4 (4.0%)
Pseudomonas species	3 (0.9%)	0 (0.0%)
Chromobacterium species	2 (0.6%)	0 (0.0%)
Chryseomonas species	2 (0.6%)	0 (0.0%)
Pseudomonas stutzeri	2 (0.6%)	0 (0.0%)
Chryseobacterium shandongense	1 (0.3%)	0 (0.0%)
Paenibacillus species	1 (0.3%)	0 (0.0%)
Pantoea species	1 (0.3%)	0 (0.0%)
Peribacillus species	1 (0.3%)	0 (0.0%)
Pseudoxanthomonas species	1 (0.3%)	0 (0.0%)
Sphingobacterium species	1 (0.3%)	0 (0.0%)
Sphingomonas koreensis	1 (0.3%)	0 (0.0%)
Sphingomonas species	1 (0.3%)	0 (0.0%)

Supplementary Table 3 Skin type microorganisms cultured from duodenoscopes in the period employing conventional cleaning compared with the period employing the AquaTYPHOON system.

Skin type microorganisms	Conventional cleaning methods (N = 333)	AquaTYPHOON system (N = 100)
Bacillus cereus	64 (19.2%)	5 (5.0%)
Bacillus species	24 (7.2%)	18 (18.0%)
Cupriavidus species	20 (6.0%)	9 (9.0%)
Micrococcus luteus	15 (4.5%)	1 (1.0%)
Cellulosimicrobium species	7 (2.1%)	6 (6.0%)
Staphylococcus warneri	10 (3.0%)	0 (0.0%)
Cellulosimicrobium cellulans	7 (2.1%)	1 (1.0%)
Gram-positive rods	8 (2.4%)	0 (0.0%)
Micrococcus species	6 (1.8%)	0 (0.0%)
Gram-variable rods	5 (1.5%)	0 (0.0%)
Staphylococcus epidermidis	5 (1.5%)	0 (0.0%)
Staphylococcus hominis	5 (1.5%)	0 (0.0%)
Bacillus simplex	4 (1.2%)	0 (0.0%)
Staphylococcus capitis	3 (0.9%)	1 (1.0%)
Brevibacterium casei	3 (0.9%)	0 (0.0%)
Staphylococcus species	3 (0.9%)	0 (0.0%)
Bacillus circulans	1 (0.3%)	0 (0.0%)
Bacillus clausii	0 (0.0%)	1 (1.0%)
Bacillus megaterium	1 (0.3%)	0 (0.0%)
Bacillus subtilis	1 (0.3%)	0 (0.0%)
Corynebacterium aurimucosum	1 (0.3%)	0 (0.0%)
Corynebacterium imitans	0 (0.0%)	1 (1.0%)
Corynebacterium mucifaciens	1 (0.3%)	0 (0.0%)
Corynebacterium tuberculostearicum	0 (0.0%)	1 (1.0%)
Corynebacterium urealyticum	0 (0.0%)	1 (1.0%)
Gram-positive cocci	1 (0.3%)	0 (0.0%)

Endosc Int Open | DOI 10.1055/a-2536-8061 | © The Author(s).
